# Iterative Diffusion-Based Distributed Cubature Gaussian Mixture Filter for Multisensor Estimation

**DOI:** 10.3390/s16101741

**Published:** 2016-10-20

**Authors:** Bin Jia, Tao Sun, Ming Xin

**Affiliations:** 1 Intelligent Fusion Technology, Germantown, MD 20876, USA; binjiaqm@gmail.com; 2 Department of Mechanical and Aerospace Engineering, University of Missouri, Columbia, MO 65211, USA; tspg5@mail.missouri.edu

**Keywords:** sensor networks, distributed estimation, Gaussian mixture, diffusion

## Abstract

In this paper, a distributed cubature Gaussian mixture filter (DCGMF) based on an iterative diffusion strategy (DCGMF-ID) is proposed for multisensor estimation and information fusion. The uncertainties are represented as Gaussian mixtures at each sensor node. A high-degree cubature Kalman filter provides accurate estimation of each Gaussian mixture component. An iterative diffusion scheme is utilized to fuse the mean and covariance of each Gaussian component obtained from each sensor node. The DCGMF-ID extends the conventional diffusion-based fusion strategy by using multiple iterative information exchanges among neighboring sensor nodes. The convergence property of the iterative diffusion is analyzed. In addition, it is shown that the convergence of the iterative diffusion can be interpreted from the information-theoretic perspective as minimization of the Kullback–Leibler divergence. The performance of the DCGMF-ID is compared with the DCGMF based on the average consensus (DCGMF-AC) and the DCGMF based on the iterative covariance intersection (DCGMF-ICI) via a maneuvering target-tracking problem using multiple sensors. The simulation results show that the DCGMF-ID has better performance than the DCGMF based on noniterative diffusion, which validates the benefit of iterative information exchanges. In addition, the DCGMF-ID outperforms the DCGMF-ICI and DCGMF-AC when the number of iterations is limited.

## 1. Introduction

With the rapid progress of the sensing and computing technologies, multiple sensors have been widely used in estimation applications, such as target tracking, wireless sensor networks, guidance and navigation, and environmental monitoring. Effective information fusion from multiple sensors is of utmost importance. It can be done in a centralized or distributed manner. For the centralized fusion, the information obtained by all sensors is collected and processed by the central node. This approach enables the global solution but requires a large amount of power and resources in communication and computation. The failure or delay on the central node may significantly degrade the estimation performance. For the distributed estimation, the information at each sensor node is processed locally and then fused to establish the global information by well-designed distributed fusion algorithms using only the local information. In contrast to the centralized estimation, the distributed estimation offers a number of advantages, such as scalability, robustness to single point of failure, low communication load, and low operation cost.

When the estimation is processed at each local sensor, it is a regular filtering problem, which has been intensively researched for decades. In many practical estimation problems, the system dynamics and measurement equations are nonlinear and the uncertainties or noises are non-Gaussian. To address this challenging filtering problem, Gaussian mixture-based filters [1] and sequential Monte Carlo-based filters [2] are two classes of widely used approaches. The rationale behind the Gaussian mixture-based filters is that any probability density function (pdf) can be approximated by the summation of a finite number of Gaussian distributions. The Monte Carlo-based filters or particle filters use a large number of particles to represent the pdf. Although some solutions have been proposed to alleviate the curse of the dimensionality problem for application of particle filters in high-dimensional problems, the computation complexity is still prohibitive. Therefore, from the computation efficiency perspective in the sensor network setting, the Gaussian mixture filter is a better alternative and will be used in this paper for multiple sensor estimation. The mean and covariance of each Gaussian component are predicted and updated using the cubature Kalman filtering (CKF) algorithm [3,4]. The fifth-degree CKF [4] is used because it is more accurate than the conventional third-degree CKF in [3] and other well-known nonlinear Gaussian filters such as the extended Kalman filter (EKF) [5] and the unscented Kalman filter (UKF) [6], which is a third-degree Gaussian filter as well.

After the local estimation is obtained at each sensor node, information fusion of the estimates from multiple sensors is conducted using the distributed estimation algorithm. Distributed estimation has been a research subject of considerable interest in the past few years [7,8,9,10,11,12,13,14,15,16,17]. Olfati-Saber [7,8] first addressed the distributed estimation problem by reducing it to two average consensus filters, one for weighted measurement and the other for information form of the covariance matrix. Because each sensor node only communicates with its immediate neighbors, the average consensus strategy is effective to obtain the average of each node’s initial value. In each iteration, each node updates its state by weighting its prior state and its neighbors’ prior states. When the number of iterations approaches infinity, average consensus can be achieved. In the consensus-based distributed estimation framework, certain requirement on the network topology is usually necessary. In [9,10], information from an individual node is propagated through the entire network via a new information-weighted consensus scheme. Although each node has limited observability of the states, even including naive agents (not having measurement), the proposed information-weighted consensus filter for distributed maximum a posterior parameter estimation and state estimation is capable of obtaining a final estimate comparable to that obtained from the centralized filter. However, it only considered the scenario that all local estimates and measurement errors are independent or uncorrelated. Sun et al. [11] proposed a batch covariance intersection technique combined with average consensus algorithms to address the correlation issue. But, the Gaussian assumption is made on all estimation processes. It may be inadequate for highly nonlinear systems and/or non-Gaussian systems. On the other hand, due to the constraints on energy and communication frequency, a large number of iterations in consensus algorithms are not feasible in practice, especially for the systems in which the time interval between two consecutive measurements is very small. 

Diffusion strategies for distributed estimation proposed in [12] overcome the disadvantage of excessive energy and communication requirements in the average consensus-based estimation. There are two steps between consecutive filtering cycle in the diffusion algorithm: incremental and diffusion. The incremental step runs a local filtering at each node with a regular time update and multiple measurement updates by incrementally incorporating measurements from every neighboring node. The diffusion step computes the ultimate fused estimate by convex combination of all estimates from the present node and its neighbors. Each node only communicates with its direct neighbors twice in each filtering cycle. The first communication collects the innovation information from its neighbors. The second communication exchanges the state estimate among neighbors from the incremental step to do the diffusion update. The estimate obtained through the diffusion strategy has been proved unbiased for linear systems. The paper [12] also provides the mean, mean square, and convergence analysis and shows that the estimate is stable under the assumption that the state space model is time invariant and each local system (joint measurement model of one node and its immediate neighbors) is detectable and stabilizable. As long as the individual node satisfies the assumption, this diffusion strategy does not have any requirement for the network topology. Diffusion recursive least-squares (RLS) algorithm was developed in [13] to deal with the distributed estimation problem and achieved the performance close to the global solution. It does not require transmission or inversion of matrices and, therefore, reduces computational complexity. It was shown that the distributed solution is asymptotically unbiased and stable if the regressors are zero-mean and temporally independent, and the inverse of covariance matrices at different time indexes can be replaced by its expected value. A diffusion least-mean-squares (LMS) algorithm was proposed in [14] with two versions: adapt-then-combine and combine-then-adapt. Mean and mean square performance were analyzed. Besides, the scheme of optimizing the diffusion LMS weights was discussed. The work of [15] extended the work in [12] by using the covariance intersection to yield a consistent estimate and relaxing the assumption made in [12]. It only requires partial local uniform observability rather than all local systems’ observability assumed in [12]. The case of no local uniform observability was discussed in [15] as well but relied on the consensus filter. Hlinka et al. [16] proposed the distributed estimation scheme using the iterative covariance intersection (ICI). Like the consensus strategy, the ICI needs recursive update of each node’s state and covariance until they converge. Each iteration can guarantee a consistent estimate. However, the ICI does not include the incremental update as the diffusion does.

Most of the aforementioned work assumes a linear dynamic process and measurement with Gaussian noise or initial uncertainty with Gaussian pdf. For highly nonlinear dynamic systems with non-Gaussian statistics, the performance of those distributed estimation methods may degrade. In this paper, we propose a new distributed Gaussian mixture filtering based on an iterative diffusion strategy to handle the distributed nonlinear estimation. There is limited literature on the distributed Gaussian mixture filtering. In [17], the likelihood consensus strategy was used in the design of a distributed Gaussian mixture filter in a sensor network that was not fully connected. Unlike the original consensus-based distributed estimation, the Gaussian mixture weight cannot be updated through the consensus filter directly since it needs to evaluate a product term of the likelihood function. By the natural logarithm transformation, the product term is transformed to a summation to which the consensus algorithm can be applied. The contributions of the proposed approach in this paper are: (1) a new distributed Gaussian mixture filtering framework with an embedded cubature rule can more accurately handle nonlinear and non-Gaussian distributed estimation problems; (2) the iterative diffusion strategy provides better fusion performance than the original diffusion method, the average consensus, and the ICI; (3) it does not need intensive communications as required in the consensus-based estimation; (4) the convergence analysis and information theoretic interpretation of the proposed approach are given.

The remainder of this paper is organized as follows. In Section 2, a centralized cubature Gaussian mixture filter is introduced. The distributed cubature Gaussian mixture filter using iterative diffusion is proposed in Section 3. In Section 4, the performance demonstration via a target-tracking problem is presented. Concluding remarks are given in Section 5.

## 2. Centralized Cubature Gaussian Mixture Filter

Consider a class of nonlinear discrete-time dynamic systems described by

(1) $$x_{k}=f\left(x_{k-1}\right)+v_{k-1}$$

(2) $$y_{k,j}=h_{j}\left(x_{k}\right)+n_{k,j}$$

where $x_{k}\in {\mathbb{R}}^{n}$ is the state vector and $y_{k,j}\in {\mathbb{R}}^{m}$ is the measurement by the *j*th sensor where the subscript “*j*” denotes the sensor index. $v_{k-1}$ and $n_{k,j}$ are the process noise and measurement noise, respectively, and their probability density functions (pdf) are represented by the Gaussian mixtures (GM) $p\left(v_{k}\right)=\sum_{p=1}^{N_{p}}\alpha^{p}N\left(v_{k}^{p};{\bar{v}}_{k}^{p},Q_{k}^{p}\right)$ and $p\left(n_{k,j}\right)=\sum_{q=1}^{N_{q}}\alpha_{j}^{q}N\left(n_{k,j}^{q};{\bar{n}}_{k,j}^{q},R_{k,j}^{q}\right)$, respectively, where $N\left(n_{k,j}^{q};{\bar{n}}_{k,j}^{q},R_{k,j}^{q}\right)$ denotes a normal distribution with mean ${\bar{n}}_{k,j}^{q}$ and covariance $R_{k,j}^{q}$ and α is the weight of the Gaussian component. The superscripts “*p*” and “*q*” denote the *p*th and *q*th component of the GM; “$N_{p}$” and “$N_{q}$” denote the number of Gaussian components. Due to the non-Gaussian noise and nonlinear dynamics, the estimated state will have a non-Gaussian pdf, which can be modeled as the GM as well.

### 2.1. Cubature Gaussian Mixture Kalman Filter

**Figure 1:**
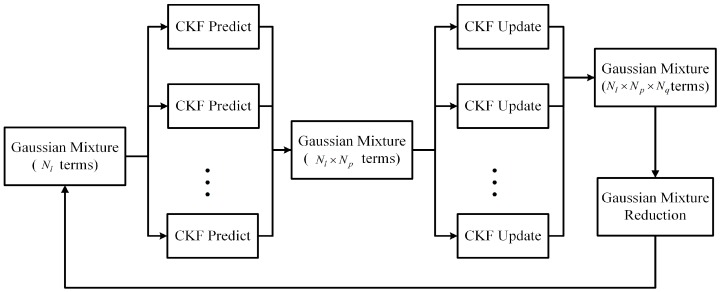
One filtering cycle of the cubature Gaussian mixture filter (CGMF).

Assume that the initial state pdf at the beginning of each filtering cycle can be represented by the GM $p\left(x\right)=\sum_{l=1}^{N_{l}}\alpha^{l}N\left(x;{\hat{x}}^{l},P^{l}\right)$. In Figure 1, one cycle of the cubature Gaussian mixture filter (CGMF) is illustrated. The cubature Kalman filter (CKF) [3,4] runs on each component of the GM to predict and update the component’s mean and covariance. The prediction step of the CKF is first used for each of the $N_{l}$ GM components. Note that after the prediction step, there are $N_{l}\times N_{p}$ Gaussian components contributed by the GM of the initial state pdf and the GM of the process noise. After that, the update step of the CKF is used for each Gaussian component and leads to $N_{l}\times N_{p}\times N_{q}$ Gaussian components added by the GM of the measurement noise. It can be seen that the number of Gaussian components increases after each filtering cycle. To limit the computational complexity, the number of Gaussian components has to be reduced after the update step. In the following, the prediction step and the update step for each Gaussian component using the CKF framework [3,4] are introduced.

#### 2.1.1. Prediction Step

Given the initial estimate of the mean ${\hat{x}}_{k-1|k-1}^{l}$ and covariance $P_{k-1|k-1}^{l}$ at time k−1 for the *l*th Gaussian component, the predicted mean and covariance can be computed by the quadrature approximation [3,4]

(3) $${\hat{x}}_{k|k-1}^{l,p}=\sum_{i=1}^{N_{u}}W_{i}f\left(\xi_{k-1,i}^{l}\right)+{\bar{v}}_{k-1}^{p}$$

(4) $$P_{k|k-1}^{l,p}=\sum_{i=1}^{N_{u}}W_{i}\left[f\left(\xi_{k-1,i}^{l}\right)-\left({\hat{x}}_{k|k-1}^{l,p}-{\bar{v}}_{k-1}^{p}\right)\right]{\left[f\left(\xi_{k-1,i}^{l}\right)-\left({\hat{x}}_{k|k-1}^{l,p}-{\bar{v}}_{k-1}^{p}\right)\right]}^{T}+Q_{k}^{p}$$

where $N_{u}$ is the total number of cubature points, $l=1,\cdots ,N_{l}$, $p=1,\cdots ,N_{p}$; The superscript “*l*,*p*” denotes the value using the *l*th Gaussian component of the GM of the initial state pdf and the *p*th component of the GM of the process noise. ${\bar{v}}_{k-1}^{p}$ is the mean of the *p*th Gaussian component of the GM representation of the process noise; $\xi_{k-1,i}^{l}$ is the transformed cubature point given by

(5) $$\xi_{k-1,i}^{l}=S_{k-1}^{l}\gamma_{i}+{\hat{x}}_{k-1|k-1}^{l},\;P_{k-1|k-1}^{l}=S_{k-1}^{l}{\left(S_{k-1}^{l}\right)}^{T}$$

The cubature points $\gamma_{i}$ and weights $W_{i}$ of the third-degree cubature rule [3] are given by

(6a) $$\gamma_{i}=\{\begin{array}{cc} \sqrt{n}e_{i} & i=1,\cdots ,n\\ -\sqrt{n}e_{i-n} & i=n+1,\cdots ,2n \end{array}$$

(6b) $$W_{i}=1/\left(2n\right),\;i=1,\cdots ,2n$$

where $e_{i}$ is a unit vector with the *i*th element being 1. In this paper, the fifth-degree cubature rule [4] is also used to improve the estimation accuracy. The weights $W_{i}$ and points $\gamma_{i}$ of the fifth-degree rule are given by

(7a) $$W_{1}=2/\left(n+2\right)$$

(7b) $$W_{i}=\frac{n^{2}\left(7-n\right)}{2{\left(n+1\right)}^{2}{\left(n+2\right)}^{2}},\;i=2,\cdots 2n+3$$

(7c) $$W_{i}=\frac{2{\left(n-1\right)}^{2}}{{\left(n+1\right)}^{2}{\left(n+2\right)}^{2}},\;i=2n+4,\cdots ,n^{2}+3n+3$$

(8a) $$\gamma_{1}=0$$

(8b) $$\gamma_{i}=\sqrt{n+2}\times {\bar{s}}_{i-1},\;i=2,\cdots ,n+2$$

(8c) $$\gamma_{i}=-\sqrt{n+2}\times {\bar{s}}_{i-n-2},\;i=n+3,\cdots ,2n+3$$

(8d) $$\gamma_{i}=\sqrt{n+2}\times {\tilde{s}}_{i-2n-3},\;i=2n+4,\cdots ,2n+3+n\left(n+1\right)/2$$

(8e) $$\gamma_{i}=-\sqrt{n+2}\times {\tilde{s}}_{i-\left(2n+3+n\left(n+1\right)/2\right)},\;i=2n+4+n\left(n+1\right)/2,\cdots ,n^{2}+3n+3$$

where the points ${\bar{s}}_{i}$ are given by

(9) $${\bar{s}}_{i}=\left[p_{i,1},p_{i,2},\cdots ,p_{i,n}\right],\;i=1,2,\cdots ,n+1$$

(10) $$p_{i,j}≜\{\begin{array}{l} -\sqrt{\frac{n+1}{n\left(n-j+2\right)\left(n-j+1\right)}}\;j<i\\ \begin{array}{l} \sqrt{\frac{\left(n+1\right)\left(n-i+1\right)}{n\left(n-i+2\right)}}\;i=j\\ 0\;j>i \end{array} \end{array}$$

and

(11) $$\left\{{\tilde{s}}_{i}\right\}≜\left\{\sqrt{\frac{n}{2\left(n-1\right)}}\left({\bar{s}}_{k}+{\bar{s}}_{l}\right):k<l;\;k,l=1,2,\cdots ,n+1\right\}$$

#### 2.1.2. Update Step

(12) $${\hat{x}}_{k|k}^{l,p,q}={\hat{x}}_{k|k-1}^{l,p}+L_{k}^{l,p,q}\left(y_{k}-z_{k}^{l,p,q}\right)$$

(13) $$P_{k|k}^{l,p,q}=P_{k|k-1}^{l,p}-L_{k}^{l,p,q}{\left(P_{xz}^{l,p,q}\right)}^{T}$$

(14) $$L_{k}^{l,p,q}=P_{xz}^{l,p,q}{\left(R_{k}^{q}+P_{zz}^{l,p,q}\right)}^{-1}$$

(15) $$z_{k}^{l,p,q}=\sum_{i=1}^{N_{u}}W_{i}h\left({\tilde{\xi }}_{k,i}^{l,p}\right)+{\bar{n}}_{k}^{q}$$

(16) $$P_{xz}^{l,p,q}=\sum_{i=1}^{N_{u}}W_{i}\left[{\tilde{\xi }}_{k,i}^{l,p}-\left({\hat{x}}_{k|k-1}^{l,p}-{\bar{v}}_{k}^{p}\right)\right]{\left[h\left({\tilde{\xi }}_{k,i}^{l,p}\right)-\left(z_{k}^{l,p,q}-{\bar{n}}_{k}^{q}\right)\right]}^{T}$$

(17) $$P_{zz}^{l,p,q}=\sum_{i=1}^{N_{u}}W_{i}\left[h\left({\tilde{\xi }}_{k,i}^{l,p}\right)-\left(z_{k}^{l,p,q}-{\bar{n}}_{k}^{q}\right)\right]{\left[h\left({\tilde{\xi }}_{k,i}^{l,p}\right)-\left(z_{k}^{l,p,q}-{\bar{n}}_{k}^{q}\right)\right]}^{T}$$

${\bar{n}}_{k}^{q}$ is the mean of the *q*th Gaussian component of the GM representation of the measurement noise; ${\tilde{\xi }}_{k,i}^{l,p}$ is the transformed cubature point given by

(18) $${\tilde{\xi }}_{k,i}^{l,p}={\tilde{S}}_{k}^{l,p}\gamma_{i}+{\hat{x}}_{k|k-1}^{l,p},\;P_{k|k-1}^{l,p}={\tilde{S}}_{k}^{l,p}{\left({\tilde{S}}_{k}^{l,p}\right)}^{T}$$

**Remark** **1:** The weight for the Gaussian component $N\left(x;{\hat{x}}_{k|k}^{l,p,q},P_{k|k}^{l,p,q}\right)$ is $\alpha^{l}\cdot \alpha^{p}\cdot \alpha^{q}$. The final GM can be represented by $\sum_{l=1}^{N_{l}}\sum_{p=1}^{N_{p}}\sum_{q=1}^{N_{q}}\alpha^{l}\alpha^{p}\alpha^{q}N\left(x;{\hat{x}}_{k|k}^{l,p,q},P_{k|k}^{l,p,q}\right)$.

Note that the number of Gaussian components increases significantly as the time evolves. In order to avoid excessive computation load, some Gaussian components can be removed or merged. There are many GM reduction algorithms [18,19,20], such as pruning Gaussian components with negligible weights, joining near Gaussian components, and regeneration of GM via Kullback–Leibler approach. In this paper, near Gaussian components are joined to reduce the number of Gaussian components. The detailed description of this method is omitted since it is not the focus of this paper and can be seen in [20]. Note that to keep the estimation accuracy, the GM reduction procedure is not necessary if the number of Gaussian components is less than a specified threshold. For the convenience of implementing the diffusion update step in the proposed distributed estimation algorithm, the number of reduced Gaussian components at each sensor node is specified a priori to be the same.

### 2.2. Centralized Cubature Gaussian Mixture Filter

The centralized cubature Gaussian mixture filter (CCGMF) can be more conveniently expressed using the information filtering form. In the information filter, the information state and the information matrix of the Gaussian component with index l,p,q at time k−1 are defined as ${\hat{y}}_{k-1|k-1}^{l,p,q}={\left(P_{k-1|k-1}^{l,p,q}\right)}^{-1}{\hat{x}}_{k-1|k-1}^{l,p,q}$ and $Y_{k-1|k-1}^{l,p,q}={\left(P_{k-1|k-1}^{l,p,q}\right)}^{-1}$, respectively. The prediction of the information state and information matrix can be obtained via Equations (3) and (4). Using the information from multiple sensors, the information state and the information matrix can be updated by [4,21]

(19) $${\hat{y}}_{k|k}^{l,p,q}={\hat{y}}_{k|k-1}^{l,p}+\sum_{j=1}^{N_{sn}}i_{k,j}^{l,p,q}$$

(20) $$Y_{k|k}^{l,p,q}=Y_{k|k-1}^{l,p}+\sum_{j=1}^{N_{sn}}I_{k,j}^{l,p,q}$$

where $N_{sn}$ is the number of sensor nodes. ${\hat{y}}_{k|k-1}^{l,p}$ and $Y_{k|k-1}^{l,p}$ can be obtained from the results of Equations (3) and (4). The information state contribution $i_{k,j}^{l,p,q}$ and the information matrix contribution $I_{k,j}^{l,p,q}$ of the *j*th sensor are given by [4,21]

(21) $$i_{k,j}^{l,p,q}={\left(P_{k|k-1}^{l,p}\right)}^{-1}P_{k|k-1,xz_{j}}^{l,p}{\left(R_{k,j}^{q}\right)}^{-1}\left[\left(y_{k,j}-z_{k,j}^{l,p,q}\right)+{\left(P_{k|k-1,xz_{j}}^{l,p}\right)}^{T}{\left(P_{k|k-1}^{l,p}\right)}^{-T}{\hat{x}}_{k|k-1}^{l,p}\right]$$

(22) $$I_{k,j}^{l,p,q}={\left(P_{k|k-1}^{l,p}\right)}^{-1}P_{k|k-1,xz_{j}}^{l,p}{\left(R_{k,j}^{q}\right)}^{-1}{\left(P_{k|k-1,xz_{j}}^{l,p}\right)}^{T}{\left(P_{k|k-1}^{l,p}\right)}^{-T}$$

Note that $z_{k,j}^{l,p,q}$ and $P_{k|k-1,xz_{j}}^{l,p}$ can be calculated by the cubature rules Equations (15) and (16), respectively, given in Section 2.1.2.

**Remark** **2:** From Equations (19) and (20), it can be seen that the local information contributions of $i_{k,j}^{l,p,q}$ and $I_{k,j}^{l,p,q}$ are only computed at sensor j and the total information contribution is simply the sum of the local contributions. Therefore, the information filter is more convenient for multiple sensor estimation than the original Kalman filter.

The CCGMF needs to know the information from all sensor nodes and thus demands a large amount of communication energy, which is prohibitive for large-scale sensor networks. In the next section, an iterative diffusion-based distributed cubature Gaussian mixture filter is proposed to provide more efficient multisensor estimation.

## 3. Iterative Diffusion-Based Distributed Cubature Gaussian Mixture Filter

The distributed estimation lets each sensor node process its local estimation and then fuse the information from its neighboring nodes by distributed estimation algorithms to establish the global estimate. In this paper, a new distributed cubature Gaussian mixture filter based on iterative diffusion (DCGMF-ID) is introduced.

The diffusion strategy is more feasible in practice when the measurement needs to be processed in a timely manner without many iterations as in the consensus algorithm. The ordinary diffusion Kalman filter (DKF) [12,13,14,15] was designed for linear estimation problems. In this paper, the new DCGMF-ID integrates the cubature rule as well as the GM into the DKF framework to address the nonlinear distributed estimation problem. The prediction step of the DCGMF-ID at each sensor node uses the cubature rule given in Section 2.1.1. The update steps of the DCGMF-ID include the incremental update and the diffusion update, which are described as follows. 

### 3.1. Incremental Update

Each node broadcasts its prediction information to its immediate neighbors and receives the prediction information from its immediate neighbors at the same time step. For every node *j*, once receiving the information, the information state and the information matrix are updated by

(23) $${\hat{y}}_{k|k,j}^{l,p,q}={\hat{y}}_{k|k-1,j}^{l,p}+\sum_{j\prime \in N_{j}}i_{k,j\prime }^{l,p,q}$$

(24) $$Y_{k|k,j}^{l,p,q}=Y_{k|k-1,j}^{l,p}+\sum_{j\prime \in N_{j}}I_{k,j\prime }^{l,p,q}$$

where $N_{j}$ denotes the set of sensor nodes containing node *j* and its immediate neighbors.

### 3.2. Diffusion Update

As mentioned in Section 2.1.2, the number of Gaussian components after the GM reduction at each node is specified a priori to be the same, for the convenience of implementing the diffusion update. The covariance intersection algorithm can be utilized for the diffusion update. The covariance for node *j* can be updated by

(25) $${\left(P_{k,j}^{l,p,q}\right)}^{-1}=\sum_{j\prime \in N_{j}}w_{j,j\prime }^{l,p,q}{\left(P_{k|k,j\prime }^{l,p,q}\right)}^{-1}$$

or in the information matrix form

(26) $$Y_{k,j}^{l,p,q}=\sum_{j\prime \in N_{j}}w_{j,j\prime }^{l,p,q}Y_{k|k,j,j\prime }^{l,p,q}$$

where $P_{k|k,j\prime }^{l,p,q}$ denotes the covariance of the j′th sensor associated with the l,p,qth Gaussian component. $w_{j,j\prime }^{l,p,q}$ is the covariance intersection weight.

The state estimation for node *j* can be updated by

(27) $${\left(P_{k,j}^{l,p,q}\right)}^{-1}{\hat{x}}_{k,j}^{l,p,q}=\sum_{j\prime \in N_{j}}w_{j,j\prime }^{l,p,q}{\left(P_{k|k,j\prime }^{l,p,q}\right)}^{-1}{\hat{x}}_{k|k,j\prime }^{l,p,q}$$

or in the information state form

(28) $${\hat{y}}_{k,j}^{l,p,q}=\sum_{j\prime \in N_{j}}w_{j,j\prime }^{l,p,q}{\hat{y}}_{k|k,j\prime }^{l,p,q}$$

The weights $w_{j,j\prime }^{l,p,q}$ are calculated by [22]

(29) $$\{\begin{array}{l} w_{j,j\prime }^{l,p,q}=\frac{1/\mathrm{tr}\left({\left(Y_{k|k,j\prime }^{l,p,q}\right)}^{-1}\right)}{\sum_{j\prime \in N_{j}}1/\mathrm{tr}\left({\left(Y_{k|k,j\prime }^{l,p,q}\right)}^{-1}\right)}\\ w_{j,j\prime }^{l,p,q}=0,\;j\prime \notin N_{j} \end{array},\;j\prime \in N_{j}$$

where tr(⋅) denotes the trace operation.

**Remark** **3:** Different from the conventional diffusion-based distributed estimation algorithms, the DCGMF-ID performs the diffusion update multiple times iteratively, rather than updating it only once. The advantage of the iterative diffusion update is that estimates from different sensors eventually converge.

The DCGMF-ID algorithm (Algorithm 1) can be summarized as follows:

**Algorithm 1****Step** **1:**Each sensor node calculates the local prediction using Equations (3) and (4), and the cubature rule, and transforms them to the information state ${\hat{y}}_{k|k-1,j}^{l,p}$ and the information matrix $Y_{k|k-1,j}^{l,p}$.**Step** **2:**When new measurements are available, each node evaluates the information state contribution $i_{k,j}^{l,p,q}$ and the information matrix contribution $I_{k,j}^{l,p,q}$ by using Equations (21) and (22).**Step** **3:**Each node communicates with its immediate neighbors to update its information state and information matrix through the incremental update (i.e., Equations (23) and (24)).**Step** **4:**Each node runs the diffusion update by Equations (26) and (28) multiple times. Let *t* denote the *t*th iteration of the diffusion update. The iterative diffusion updates can be given by
(30a)$${\hat{y}}_{k|k,j}^{l,p,q}\left(t+1\right)=\sum_{j\prime \in N_{j}}w_{j,j\prime }^{l,p,q}\left(t\right){\hat{y}}_{k|k,j\prime }^{l,p,q}\left(t\right)$$
(30b)$$Y_{k|k,j}^{l,p,q}\left(t+1\right)=\sum_{j\prime \in N_{j}}w_{j,j\prime }^{l,p,q}(t)Y_{k|k,j\prime }^{l,p,q}(t)$$
When $t=t_{\max}$, the final estimates are $Y_{k,j}^{l,p,q}=Y_{k|k,j}^{l,p,q}\left(t_{\max}\right)$; ${\hat{y}}_{k,j}^{l,p,q}={\hat{y}}_{k|k,j}^{l,p,q}\left(t_{\max}\right)$Calculate the mean ${\hat{x}}_{k|k}^{l,p,q}$ and covariance $P_{k|k}^{l,p,q}$ of each Gaussian component.The final GM can be represented by $\sum_{l=1}^{N_{l}}\sum_{p=1}^{N_{p}}\sum_{q=1}^{N_{q}}\alpha^{l}\alpha^{p}\alpha^{q}N\left(x;{\hat{x}}_{k|k}^{l,p,q},P_{k|k}^{l,p,q}\right)$.**Step** **5:**Conduct GM reduction.**Step** **6:**Let k=k+1; continues to Step 1.

The iterative diffusion update is identical to the iterative covariance intersection (ICI) algorithm [16]. Thus, the proposed distributed estimation has the same properties of unbiasedness and consistency as the ICI. For linear systems, if the initial estimate at each sensor node is unbiased, the estimate through the incremental update and the diffusion update in each filtering cycle is still unbiased. For nonlinear systems, however, the unbiasedness may not be preserved. It is also true for the analysis of consistency. When the covariance intersection (CI) method is used for data fusion, consistency is ensured based on the assumption that the estimate at each sensor node is consistent [23]. If it is assumed that each node’s local estimate after the incremental step is consistent (i.e., $P_{k|k,j}\geq E\left[\left({\hat{x}}_{k|k,j}-x_{k}\right){\left({\hat{x}}_{k|k,j}-x_{k}\right)}^{T}\right]$), then by the diffusion update, the fused estimate is still consistent because the CI is applied. Without this assumption, consistency is not guaranteed by the CI technique. For linear systems, this assumption can be easily met and consistency can be guaranteed. For nonlinear systems, the high-degree (fifth-degree) cubature rule based-filtering is utilized in this paper for the local estimation at each node. It can provide more accurate estimate of ${\hat{x}}_{k|k,j}$ and $P_{k|k,j}$ than the third-degree cubature Kalman filter (CKF) and the unscented Kalman filter (UKF). Therefore, although the unbiasedness and consistency cannot be guaranteed for nonlinear systems, they can be better approached by the proposed distributed estimation scheme than other distributed nonlinear filters. 

It is necessary to compare the DCGMF-ID with the consensus-based distributed estimation. For the iterative diffusion strategy in the DCGMF-ID, if the local estimate obtained at each node after the incremental update is consistent, the fused estimate by the diffusion update is also consistent, no matter how many iterations of the iterative diffusion update since the CI is applied. In addition, it was shown in [16] that the covariance and estimate from each node converge to a common value (i.e., $\underset{t\to \infty }{\lim}P_{k,j}(t)=P_{k}$ and $\underset{t\to \infty }{\lim}{\hat{x}}_{k,j}(t)={\hat{x}}_{k}$). Recall that “*t*” represents the *t*th diffusion iteration, not the time. However, for the consensus-based distributed estimation [24], even if the local estimate obtained at each node is consistent, if the number of iterations of consensus is not infinite, the consistency of the fused estimate cannot be preserved [24]. Because the average consensus cannot be achieved in a few iterations, a multiplication by |N|, the cardinality of the network, will lead to an overestimate of the information, which is not desirable. Although another approach was proposed in [24]—to fuse the information from each node in order to preserve consistency—the new consensus algorithm results in more computation complexity.

In the following, we provide a more complete analysis of the convergence by the following two propositions.

**Proposition** **1:** *The iterative diffusion update Equations (30a) and (30b) can be represented in a general form of η(t+1)=A(t)η(t), where each (j,j′) entry of the transition matrix A(t) denoted by $a_{j,j\prime }(t)$ corresponds to the weight $w_{j,j\prime }^{l,p,q}\left(t\right)$. Assume that the sensor network is connected. If there exists a positive constant α<1 and the following three conditions are satisfied*

*(a)*  $a_{j,j}(t)\geq \alpha$ *for all* j,t*;*
*(b)*  $a_{j,j\prime }(t)\in \left\{0\right\}\cup [\alpha ,1]$*,* j≠j′*;*
*(c)*  $\sum_{j\prime =1}^{N_{sn}}a_{j,j\prime }(t)=1$ *for all* j,j′,t*;*

*the estimates using the proposed DCGMF-ID reach a consensus value.*

**Proof:**  The proof uses the theorem 2.4 in [25]. If the connected sensor network satisfies these three conditions, η(t), using the algorithm:

(31) η(t+1)=A(t)η(t)

converges to a consensus value. For the scalar case (the dimension of the state is one), $a_{j,j\prime }(t)$ corresponds to $w_{j,j\prime }^{l,p,q}\left(t\right)$. The jth element of η(t) corresponds to the information state ${\hat{y}}_{k|k,j}^{l,p,q}\left(t\right)$. For the vector case, the transition matrix $A(t)⊗I_{n}$ should be applied where ⊗ denotes the Kronecker product and *n* is the dimension of the state. For the matrix case, each column of the matrix can be treated as the vector case. ☐

As seen from Equation (29), the weight $w_{j,j\prime }^{l,p,q}\left(t\right)$ only depends on the covariance matrix. Here we assume that the covariance in the first iteration is upper bounded, and for any t there is no covariance matrix equal to 0 (no uncertainty). As long as node j and node j′ are connected, $w_{j,j\prime }^{l,p,q}\left(t\right)\in \left(0,1\right)$. Thus, condition (b) is satisfied. In addition, from Equation (29), $\sum_{j\prime =1}^{N_{sn}}w_{j,j\prime }^{l,p,q}\left(t\right)=1$ always holds; that is, the transition matrix A(t) is always row-stochastic. Therefore, condition (c) is satisfied.

For any arbitrary large *t*, say $t_{\max}$, the non-zero weight set $\left\{w_{j,j\prime }^{l,p,q}\left(t\right),t=1,\cdots ,t_{\max}\right\}$ for all j,j′ is a finite set since the number of nodes and the number of iterations are finite. There always exists a minimum value in this finite set. Thus, α can be chosen to be $0<\alpha \leq \min\left\{w_{j,j\prime }^{l,p,q}\left(t\right)\right\}$ such that conditions (a) and (b) are satisfied.

According to the theorem 2.4 in [25] for the agreement algorithm Equation (31), the estimate η(t) reaches a consensus value.

**Proposition** **2:** If the assumption and conditions in Proposition 1 are satisfied, the consensus estimate using the DCGMF-ID is unique.

**Proof:**  Let $U_{0,t}=A(t)A(t-1)\cdots A(0)$ be the backward product of the transition matrices and $\underset{t\to \infty }{\lim}U_{0,t}=U^{*}$ according to Proposition 1. On the other hand, when the consensus is achieved, the covariance matrix or the information matrix $Y_{k|k,j\prime }^{l,p,q}$ associated with each node becomes the same. According to Equation (29), the weights $w_{j,j\prime }^{l,p,q}\left(t\right)$ converge to the same value. Thus, $\underset{t\to \infty }{\lim}A(t)=A^{*}$ and $A^{*}1=1$ since $A^{*}$ is a row-stochastic matrix where $A^{*}={\left[a_{1}\;a_{2}\;\cdots a_{n}\right]}^{T}$ with $a_{j}$ being the row vector of the matrix $A^{*}$. Furthermore, because $Y_{k|k,j\prime }^{l,p,q}$ converges to the same value, from Equation (29), all the non-zero weights $w_{j,j\prime }^{l,p,q}\left(t\right)$ or all non-zero entries of the row vector $a_{j}$ are identical and equal to the reciprocal of the degree of the *j*th node, i.e., $\frac{1}{\delta_{j}}$ (where $\delta_{j}≜$ degree of the *j*th node ≜ cardinality of $N_{j}$ ). Hence, $A^{*}$ is deterministic given the connected sensor network. ☐

$A^{*}$ is irreducible since the sensor network is connected. Moreover, the diagonal elements of $A^{*}$ are all positive (equal to the reciprocal of the degree of each node). Hence, 1 is a unique maximum eigenvalue of $A^{*}$ [26] and, in fact, $A^{*}$ is a primitive matrix [26].

In the sense of consensus, $\underset{t\to \infty }{\lim}\eta (t)=U^{*}\eta (0)$, we have $A^{*}U^{*}=U^{*}$ or $(A^{*}-I)U^{*}=0$ (note, it is not possible for $U^{*}$ to be 0 since it is the backward product of non-negative matrices). The column of $U^{*}$ belongs to the null space of $A^{*}-I$. Since 1 is the unique maximum eigenvalue of $A^{*}$, 0 is the unique eigenvalue of $A^{*}-I$ and the dimension of the null space of $A^{*}-I$ is 1. Thus, 1 (or any scalar multiplication of 1) is the unique vector belonging to the null space of $A^{*}-I$. Therefore, $U^{*}$ is ergodic, i.e., $U^{*}=1\left[\alpha_{1},\;\alpha_{2},\cdots ,\alpha_{n}\right]$ where $\alpha_{i}$ is a scalar constant. According to Theorem 4.20 in [27], $\left[\alpha_{1},\;\alpha_{2},\cdots ,\alpha_{n}\right]$ and the consensus value of η(t) are unique.

The iterative diffusion update in the DCGMF-ID can be interpreted from the information theory perspective as the process of minimizing the Kullback–Leibler divergence (KLD) [28]. In the information theory, a measure of distance between different pdfs can be given by the KLD. Given the local pdf $p_{i}$ with the weight $\pi_{i}$, the fused pdf $p_{f}$ can be obtained by minimizing the KLD:

(32) $$p_{f}=\arg\underset{p}{\min}\sum_{i=1}^{N_{sn}}\pi_{i}D(p||p_{i})$$

with $\sum_{i=1}^{N_{sn}}\pi_{i}=1$ and $\pi_{i}\geq 0$. $D(p||p_{i})$ is the KLD defined as:

(33) $$D(p||p_{i})=\int p(x)\log\frac{p(x)}{p_{i}(x)}dx$$

The KLD is always non-negative, and equal to zero only when $p(x)=p_{i}(x)$.

The solution to Equation (32) turns out to be [28]

(34) $$p_{f}(x)=\frac{\prod_{i=1}^{N_{sn}}{\left[p_{i}(x)\right]}^{\pi_{i}}}{\int \prod_{i=1}^{N_{sn}}{\left[p_{i}(x)\right]}^{\pi_{i}}dx}$$

The above equation is also the Chernoff fusion [29]. Under the Gaussian assumption, which is true for each component of the GM model in this paper, it was shown in [29] that the Chernoff fusion yields update equations identical to the covariance intersection Equations (25)–(28).

Therefore, from the information-theoretic perspective, the iterative diffusion update Equation (30) is actually equivalent to minimizing the KLD repeatedly. For instance, the diffusion update at the *t*th iteration is equivalent to

(35) $$p_{f,j}(t+1)=\arg\underset{p_{j}(t+1)}{\min}\sum_{j\prime \in N_{j}}\omega_{j,j\prime }(t)D(p_{j}(t+1)||p_{j\prime }(t))\text{ with }j=1,\ldots ,N_{sn}$$

When *t* approaches $t_{\max}$, from the convergence property of the iterative diffusion (i.e., Propositions 1 and 2), the cost for the minimization problem in Equation (35) approaches 0 since $p_{j}(t_{\max})=\bar{p}$ for all $j=1,\ldots ,N_{sn}$, and $D(\bar{p}||\bar{p})=0$ where $\bar{p}$ is the final convergent pdf. 

## 4. Numerical Results and Analysis

In this section, the performance of DCGMF based on different fusion strategies is demonstrated via a benchmark target-tracking problem using multiple sensors, which is to track a target executing a maneuvering turn in a two-dimensional space with unknown and time-varying turn rate [3]. The target dynamics is highly nonlinear due to the unknown turn rate. It has been used as a benchmark problem to test the performance of different nonlinear filters [3,30].

The discrete-time dynamic equation of the target motion is given by:

(36) $$x_{k}=\left[\begin{array}{ccccc} 1 & \frac{\sin\left(\omega_{k-1}\Delta t\right)}{\omega_{k-1}} & 0 & \frac{\cos\left(\omega_{k-1}\Delta t\right)-1}{\omega_{k-1}} & 0\\ 0 & \cos\left(\omega_{k-1}\Delta t\right) & 0 & -\sin\left(\omega_{k-1}\Delta t\right) & 0\\ 0 & \frac{1-\cos\left(\omega_{k-1}\Delta t\right)}{\omega_{k-1}} & 1 & \frac{\sin\left(\omega_{k-1}\Delta t\right)}{\omega_{k-1}} & 0\\ 0 & \sin\left(\omega_{k-1}\Delta t\right) & 0 & \cos\left(\omega_{k-1}\Delta t\right) & 0\\ 0 & 0 & 0 & 0 & 1 \end{array}\right]x_{k-1}+v_{k-1}$$

where $x_{k}={\left[x_{k},{\dot{x}}_{k},y_{k},{\dot{y}}_{k},\omega_{k}\right]}^{T}$; $\left[x_{k},y_{k}\right]$ and $\left[{\dot{x}}_{k},{\dot{y}}_{k}\right]$ are the position and velocity at time k, respectively; Δt is the time-interval between two consecutive measurements; $\omega_{k-1}$ is the unknown turn rate at the time k−1; and $v_{k-1}$ is the white Gaussian noise with mean zero and covariance $Q_{k-1}$,

(37) $$Q_{k-1}=\left[\begin{array}{ccccc} \frac{\Delta t^{3}}{3} & \frac{\Delta t^{2}}{2} & 0 & 0 & 0\\ \frac{\Delta t^{2}}{2} & \Delta t & 0 & 0 & 0\\ 0 & 0 & \frac{\Delta t^{3}}{3} & \frac{\Delta t^{2}}{2} & 0\\ 0 & 0 & \frac{\Delta t^{2}}{2} & \Delta t & 0\\ 0 & 0 & 0 & 0 & 1.75\times 10^{-4}\Delta t \end{array}\right]$$

**Figure 2:**
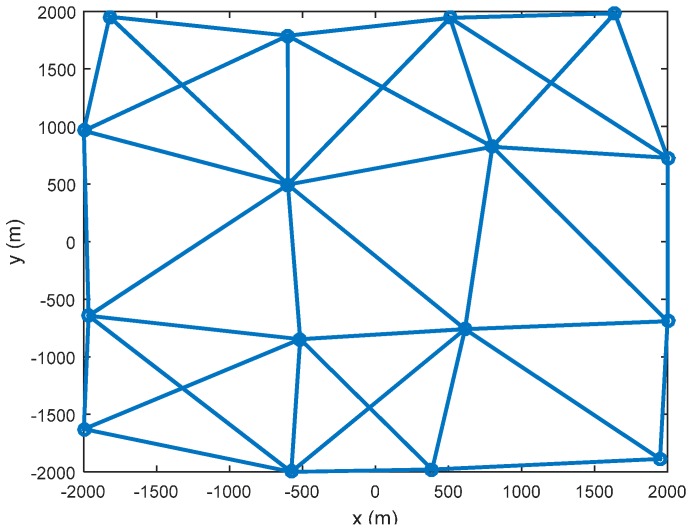
The network of sensors.

The measurements are the range and angle given by

(38) $$y_{k}=\left(\begin{array}{c} \sqrt{x_{k}^{2}+y_{k}^{2}}\\ \mathrm{atan}2\left(y_{k},x_{k}\right) \end{array}\right)+n_{k}$$

where atan2 is the four-quadrant inverse tangent function; $n_{k}$ is the measurement noise with an assumed non-Gaussian distribution $n_{k}∼0.5N\left({\bar{n}}_{1},R_{1}\right)+0.5N\left({\bar{n}}_{2},R_{2}\right)$, where ${\bar{n}}_{1}={\left[5\;m,-2\times 10^{-6}\;mrad\right]}^{T}$ and ${\bar{n}}_{2}={\left[-5\;m,0\;mrad\right]}^{T}$. The variances $R_{1}$ and $R_{2}$ are assumed to be $R_{1}=\mathrm{diag}\left(\left[100\;m^{2},10\;mrad^{2}\right]\right)$ and $R_{2}=\left[\begin{array}{cc} 80\;m^{2} & 10^{-1}\;mmrad\\ 10^{-1}\;mmrad & 10\;mrad^{2} \end{array}\right]$. The sampling interval is Δt=1 s. The simulation results are based on 100 Monte Carlo runs. The initial estimate ${\hat{x}}_{0}$ is generated randomly from the normal distribution $N\left({\hat{x}}_{0};x_{0},P_{0}\right)$ with $x_{0}$ being the true initial state $x_{0}={\left[1000\;m,300\;m/s,1000\;m,0,-3\;\deg/s\right]}^{T}$ and $P_{0}$ being the initial covariance $P_{0}=\mathrm{diag}\left(\left[100\;m^{2},10\;m^{2}/s^{2},100\;m^{2},10\;m^{2}/s^{2},100\;m{\mathrm{rad}}^{2}/s^{2}\right]\right)$. Sixteen sensors are used in simulation. The topology of the sensor network is shown in Figure 2. Note that the “circle” denotes the sensor node. It is assumed that the target is always in the range and field of view of all sensors.

**Figure 3:**
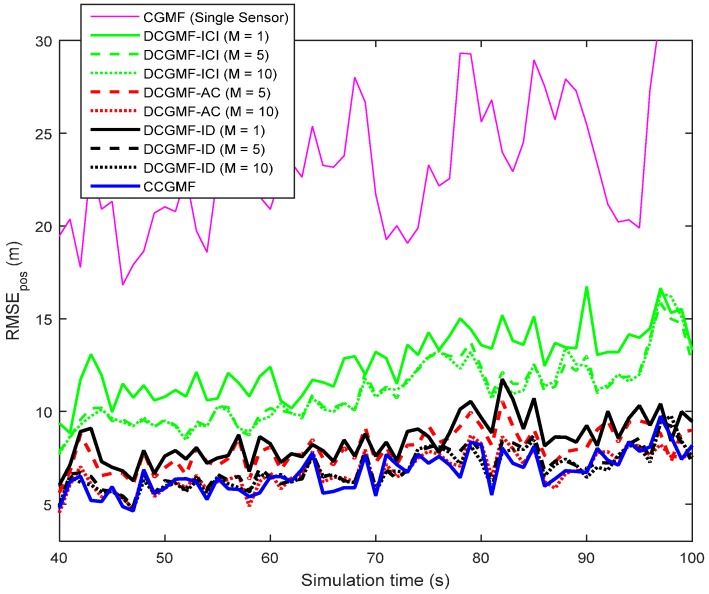
Root mean square errors (RMSEs) of the position estimation.

**Figure 4:**
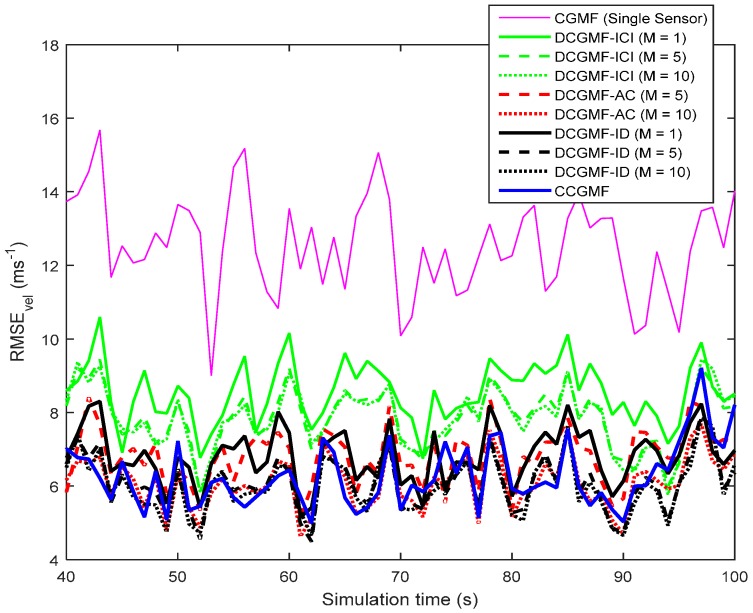
RMSEs of the velocity estimation.

**Figure 5:**
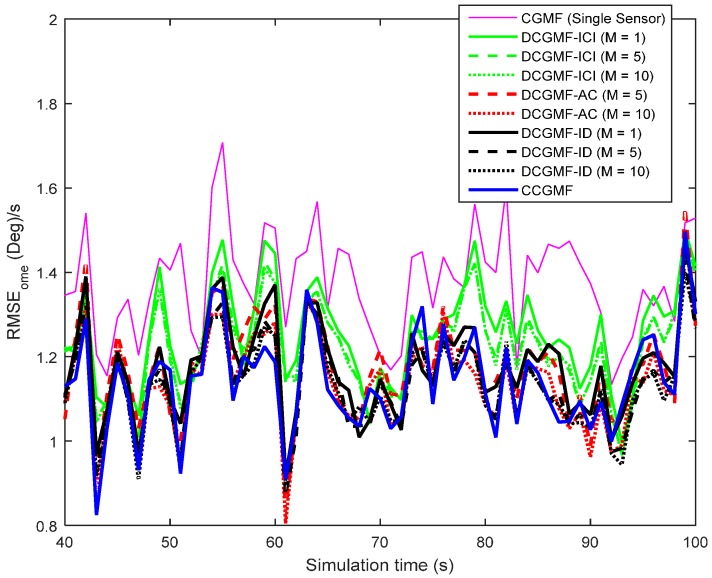
RMSEs of the turn-rate estimation.

The metric used to compare the performance of different filters is the root mean square error (RMSE). The RMSEs of the position, velocity, and turn rate using different filters with the third-degree cubature rule are shown in Figure 3, Figure 4 and Figure 5, respectively. The cubature Gaussian mixture filter (CGMF) using a single sensor, the distributed cubature Gaussian mixture filter based on the iterative covariance intersection [16] (DCGMF-ICI), average consensus (DCGMF-AC), iterative diffusion strategies (DCGMF-ID), and the centralized cubature Gaussian mixture filter (CCGMF) are compared. Since DCGMF-ICI, DCGMF-AC, and DCGMF-ID all involve iterations, it is more illustrative to use the number of iterations as a parameter to compare their performance. “M” in the figures is the iteration number. 

It can be seen from the figures that (1) DCGMFs and CCGMF exhibit better performance than CGMF using single sensor since more information from multiple sensors can be exploited; (2) with the increase of iterations, the performance of all DCGMFs is improved; (3) the DCGMF-ICI is less accurate than the DCGMF-AC and the DCGMF-ID since the ICI algorithm does not do the incremental update; (4) both the DCGMF-AC (*M* = 10) and the DCGMF-ID (*M* = 10) achieve very close performance to the CCGMF. However, fewer iterations have a more negative effect on the performance of the DCGMF-AC than that on the DCGMF-ID. The DCGMF-ID is more effective in terms of iterations since the DCGMF-ID with *M* = 1 has close performance to the DCGMF-AC with *M* = 5. Hence, when the allowable number of information exchanges is limited, DCGMF-ID would be the best filter. It is also worth noting that the DCGMF-AC requires less computational effort at each node, but requires more communication expense than the DCGMF-ID. If the communication capability of the sensor network is not a main constraint, the DCGMF-AC would be a competitive approach.

Next, we compare the performance of DCGMFs using the third-degree cubature rule and the DCGMFs using the fifth-degree cubature rule. The metric is the averaged cumulative RMSE (CRMSE). The CRMSE for the position is defined by

(39) $${\mathrm{CRMSE}}_{\mathrm{pos}}=\frac{1}{N_{mc}}\sum_{m=1}^{N_{mc}}\sqrt{\frac{1}{N_{sim}}\sum_{i=1}^{N_{sim}}\sum_{j=1,3}{\left(x_{i,m}^{j}-{\hat{x}}_{i,m}^{j}\right)}^{2}}$$

where $N_{sim}$ = 100 *s* is the simulation time and $N_{mc}=100$ is the number of Monte Carlo runs. The superscript “*j*” denotes the *j*th state variable and the subscripts “*i*” and “*m*” denote the *i*th simulation time step and the *m*th simulation, respectively. The CRMSE for the velocity and CRMSE for the turn rate can be similarly defined. 

The results of DCGMF-AC using the third-degree cubature rule and the fifth-degree cubature rule show indistinguishable difference. Similar results can be observed for CCGMF. DCGMF-ID and DCGMF-ICI using the fifth-degree cubature rule, however, show better performance than those using the third-degree cubature rule. The reason is that DCGMF-ID and DCGMF-ICI depend heavily on the local measurement to perform estimation, while DCGMF-AC and CCGMF update the estimate based on global observations from all sensors. Specifically, for DCGMF-AC, although each sensor communicates measurement only with its neighbors, after convergence of the consensus iterations, each sensor actually obtained a fused measurement information from all sensors. Because the high degree numerical rule affects the accuracy of estimates extracted from the observations, the fifth-degree cubature rule can more noticeably improve the performance of DCGMF-ID and DCGMF-ICI based on only local observations. However, the benefit of using the high-degree numerical rule will be mitigated if more information from more sensors is available as for the DCGMF-AC and CCGMF. Hence, we only compare the results of DCGMF-ID and DCGMF-ICI using the third-degree and the fifth-degree cubature rules in Table 1. In order to see merely the effect of the cubature rules with different accuracy degrees on the performance, we want to minimize the effect of different iterations on the performance of different filters. Therefore, a sufficiently large iteration number, *M* = 20, is used to ensure that the different filters already converge after iterations. It can be seen from the Table 1 that DCGMF-ID and DCGMF-ICI using the fifth-degree cubature rule can achieve better performance than those using the third-degree cubature rule. 

## 5. Conclusions 

A new iterative diffusion-based distributed cubature Gaussian mixture filter (DCGMF-ID) was proposed for the nonlinear non-Gaussian estimation using multiple sensors. The convergence property of the DCGMF-ID was analyzed. It has been shown via a target-tracking problem that the DCGMF-ID can successfully approximate the performance of the centralized cubature Gaussian mixture filter and has all the advantages of the distributed filters. Among the iterative distributed estimation strategies, the DCGMF-ID exhibits more accurate results than the iterative covariance intersection based method (i.e., DCGMF-ICI). It also shows better performance than the average consensus-based method given the same number of iterations. In addition, the fifth-degree cubature rule can improve the accuracy of the DCGMF-ID. 

## Figures and Tables

**Table 1:** Cumulative root mean square errors (CRMSEs) of different filters.

Filters	CRMSE (Position)	CRMSE (Velocity)	CRMSE (Turn Rate)
DCGMF-ID (third-degree, *M* = 20)	5.85892	5.60166	0.019392
DCGMF-ID (fifth-degree, *M* = 20)	5.78748	5.57730	0.019375
DCGMF-ICI (third-degree, *M* = 20)	8.81274	7.22025	0.020837
DCGMF-ICI (fifth-degree, *M* = 20)	8.02142	7.11939	0.020804

Distributed cubature Gaussian mixture filter based on an iterative diffusion strategy (DCGMF-ID) and DCGMF based on the iterative covariance intersection (DCGMF-ICI).
